# Iodide of Ammonium in Asthma

**Published:** 1888-07

**Authors:** 


**Iodide of Ammonium in Asthma.**—Mrs. W. came into my office for medicine for her daughter, who was suffering from "evening headache," as she expressed it—commencing about 6 p. m. and lasting until after midnight. My ℞ was, iodide of amonium ʒij. water ℥iv.; teaspoonful three times daily. This was continued until she had taken six times the quantity. Last time she called she said, "My daughter has no more headache, and she is also free from asthma: your medicine has cured both. I made a note of it, and here you have it. I trust others who have opportunities to test the merits of this drug in asthma will do so and report. It may not prove curat.ve in all cases, but by carefully tabulating the pathological conditions in each case, we may be able to pick out our case, and write "specific" after iodide of amonium.—*Dr. Cook in Eclectic Medical Journal.*

